# Characterizing User Experiences With an SMS Text Messaging–Based mHealth Intervention: Mixed Methods Study

**DOI:** 10.2196/35699

**Published:** 2022-05-03

**Authors:** Sayde Leya King, Jana Lebert, Lacey Anne Karpisek, Amelia Phillips, Tempestt Neal, Kristin Kosyluk

**Affiliations:** 1 Department of Computer Science and Engineering College of Engineering University of South Florida Tampa, FL United States; 2 Department of Mental Health Law and Policy Louis de la Parte Florida Mental Health Institute University of South Florida Tampa, FL United States; 3 Department of Child and Family Studies College of Behavioral and Community Sciences University of South Florida Tampa, FL United States; 4 Department of Medical Education Morsani College of Medicine University of South Florida Tampa, FL United States

**Keywords:** text messaging, SMS, mobile health, mHealth, stigma, user perceptions, ubiquitous sensing, low-intensity intervention, coping, mental health, cognitive restructuring, mobile phone

## Abstract

**Background:**

Limited access to mental health care services due to provider shortages, geographic limitations, and cost has driven the area of mobile health (mHealth) care to address these access gaps. Reports from the Cohen Veterans Network and National Council for Behavioral Health show that in states where mental health care is more accessible, 38% of people still do not receive the care they need. mHealth strategies help to provide care to individuals experiencing these barriers at lower cost and greater convenience, making mHealth a great resource to bridge the gaps.

**Objective:**

We present a mixed methods study to evaluate user experiences with the mental mHealth service, Cope Notes. Specifically, we aimed to investigate the following research questions: How do users perceive the service in relation to stigma, impact of the intervention, and perceived usefulness? How do users rate the Cope Notes service and SMS text messaging along various dimensions of acceptability? What is the relationship between Cope Notes SMS text message ratings, user personality, and coping strategies? What are user perspectives of leveraging ubiquitous sensing technologies to improve delivery and provide tailored content?

**Methods:**

We performed qualitative interviews with Cope Notes users (N=14) who have used the service for at least 30 days to evaluate their experiences and usefulness of the service. These interviews were coded by 2 raters (SLK and JL), and the interrater reliability was calculated with SPSS (IBM Corp) at 61.8%. In addition, participants completed quantitative measures, including a user experiences survey, personality inventory (Big Five Inventory-10), and coping assessment (Brief Coping Orientation to Problems Experienced).

**Results:**

We derived 7 themes from our qualitative interviews: Likes or Perceived Benefits, Dislikes or Limitations, Suggested Changes, Stigma or Help Seeking, Perceptions of Ubiquitous Sensing, Cultural Sensitivity, and Alternative mHealth Resources. Exploratory analyses between acceptability ratings of Cope Notes and personality factors showed statistically significant positive relationships between seeing oneself as someone who is generally trusting and acceptability items, the most significant being item 7 (*I fully understood the sentiment behind Cope Notes Messages*) with (*r*_s(10)_=0.82, *P*=.001). We also found statistically significant relationships between acceptability and Brief Coping Orientation to Problems Experienced items, with the strongest positive correlation between participants strongly endorsing coping by accepting the reality that an event has happened and acceptability item 7 (*r*_s(8)_=0.86, *P*=.001).

**Conclusions:**

Our study found that Cope Notes subscribers appreciate the service for reframing their mental wellness with statistically significant correlations between personality and acceptability of the service. We found that some users prefer a more personalized experience with neutral to positive reactions to a potential companion app that continuously monitors user behavior via smartphone sensors to provide just-in-time interventions when users need it most.

## Introduction

### Limited Access to Care

Mental health service access is scarce for many populations across the globe [1]. With many communities experiencing mental health provider shortages, funding limitations, geographic isolation, and stigma toward seeking mental health services, mobile phone interventions enhance service access due to their convenience, lower cost, and privacy [2]. At the National Institute of Mental Health’s 2018 Mental Health Services Research Conference, Dr. David Mohr of Northwestern University’s Feinberg School of Medicine declared mobile phone interventions “the next big thing” for research regarding mental health services [3]. Although specific mobile interventions, such as mobile health (mHealth) apps and SMS text message systems, have shown to be effective in enhancing access to mental health services [4], a host of unresolved questions remain, including potential impacts on stigma, user acceptability, and other technological components such as ubiquitous sensing technologies that aim to automate emotion recognition in real time without the user’s intervention. Identifying what is known and resolving what is unknown about these topics is critical in guiding next steps in research.

### Understanding the Problem: Mental Health Treatment Gap

The mental health treatment gap is a well-known phenomenon for stakeholders, especially researchers, providers, and consumers. The World Health Organization reports that “the gap between people needing care and those with access to care remains substantial, and coverage for effective treatment remains extremely low” [5]. Furthermore, this trend is far reaching; worldwide, 76% to 85% of the individuals with mental health disorders do not receive the treatment they need, particularly in low- and middle-income countries [6]. Similarly, the Cohen Veterans Network and National Council for Mental Wellbeing found that although nearly 56% of American adults seek treatment for themselves or others, 38% of American adults must wait over a week to receive services [7]. They also found that even in states where services are more accessible, >38% of people do not receive the services they need. These alarming statistics are attributed to a variety of barriers to care that must be resolved to improve service access.

### Barriers to Care

As outlined by the World Health Organization, coverage for mental health services remains a global concern [5]. According to the 2016 Commonwealth Fund’s Annual International Health Policy Survey, 15% of US adults in need of mental health services could not afford them, and these rates were similar in France (21%) and Norway (16%) [8]. Studies similarly indicated poor insurance coverage and cost as the top barrier to care for >42% of the population [7,9]. Data for low- and middle-income countries suggest that mental health expenditure across all countries is US $2 per year capita, and low-income countries report it as <25 cents [10]. The sources of funding for mental health services in low- and middle-income countries occur in the following order: *out-of-pocket expenditure by patient or family, taxes, social insurance,* and *private insurance* [11,12]. Furthermore, these data underscore the funding concerns across low- and high-income countries and highlight the importance of enhancing access to lower cost mental health interventions on a global scale.

An additional challenge that dissuades service access is the limited availability of mental health providers. The World Health Organization reports that the average ratio of mental health providers to need is approximately 9 per 100,000 [13]. This ratio is especially problematic given 1-in-7 people worldwide have one or more mental health condition [14]. In all, 77% of the counties in the United States experience a severe shortage of mental health providers, and the demand for providers is estimated to increase during and after the COVID-19 pandemic [15]. More specifically, the Health Resources and Services Administration [16] shares that the “United States is reported to have a shortage of over 10,000 full time mental health practitioners (psychiatrists; clinical, counseling, and school psychologists; substance abuse and behavioral disorder counselors; mental health and substance abuse social workers; mental health counselors; school counselors) by the year 2025.” This severe deficit in mental health practitioners may lead to other access issues, such as long wait lists, billing restrictions, transportation challenges, a lack of culturally competent care, and a lack of anonymity, all which are outlined as problematic for rural settings in particular [17]. This manuscript discusses the alternative of mHealth interventions as a viable solution to such barriers to care.

### mHealth Interventions: A Potential Solution

First coined approximately 10 years ago, mHealth interventions refer to the delivery of health services via mobile or wireless devices [18]. Common mobile mental health interventions are in the form of mobile apps, but can also include SMS text message components [19] and ubiquitous sensing [20]. SMS text message interventions rely on SMS text messages, which are a form of mobile communication that is fast, reliable, efficient, and highly accessible to the general population. Ubiquitous sensing refers to the use of wireless sensors that are embedded in mobile phones to continuously monitor the activities of mobile phone users to infer, for example, the user’s emotional or mental state [21]. In the context of mental health mHealth interventions, ubiquitous sensing can be particularly useful in the delivery of SMS text messages as it could enable delivery of content when users need them most. For example, inertial sensors (motion and force), physiological sensors (heart rate or dermal activity), and ambient sensors (light) have been shown to capture data indicative of mood changes [22], which could be leveraged for prompt delivery of just-in-time text message interventions (TMIs) [23]. A recent study evaluating a TMI found the approach to be a feasible and acceptable method of delivering psychological treatments [24]. Feasibility and acceptability are 2 implementation outcome measures that help researchers understand if a service is satisfactory (acceptable) or successfully usable (feasible) [25]. Feasibility is a criterion that relates to the practicality, whereas acceptability is a criterion relating to personal judgment [25]. In the context of mHealth interventions, feasibility may examine the ease in performance of an app, and acceptability may examine if the intervention fits an individual’s needs. Examining such measures can help facilitate maintenance of TMI and ubiquitous mHealth interventions. Given >20 billion SMS text messages are sent out every day worldwide [26] and nearly 33% of Americans reported to prefer text messaging to all other forms of communication [27], the investigation of SMS text messaging and mHealth apps, their implementation outcome measures, and their impact on mental health outcomes is well justified.

### Understanding the Effectiveness of mHealth Interventions

Despite SMS text messaging being a rather new modality for mHealth, studies have already demonstrated the positive impact of TMIs on health behaviors on a global scale. An instance of this is Text4Mood, a TMI service that sends supportive text messages written by mental health therapists daily to subscribers exclusively in Alberta, Canada. This service provides immediate access to an intervention for patients who may have limited access to care, free of charge. After surveying >4000 subscribers, >80% of respondents felt Text4Mood increased hope of managing daily issues, 77% improved management of depression and anxiety, and 75% felt more connected to a support system. Overall, 83% of participants felt Text4Mood improved their general well-being [24]. Although these outcomes suggest the effectiveness of TMI as interventions, the influence of ubiquitous sensing on such mHealth intervention outcomes is less studied. Research in this area also indicates challenges with poor study quality and reproducibility, variability of data types, characteristics of participants, environments, and privacy [28]. As a result, we present these sensors and additional service delivery components, such as the implementation outcome measures, acceptability, and usability, to the participants of this study to gain insight on perceptions as it relates to the app Cope Notes, an SMS text message–based mHealth resource, as a just-in-time intervention.

### Overview of Cope Notes

Cope Notes is an SMS text message–based support program that sends daily messages to a subscriber [29]. Currently, these text messages are sent daily at a random time with the aim of increasing the recipient’s mental wellness and providing peer support. The goal of these messages is to increase positive thought patterns and build healthy emotional tendencies of subscribers. This program resembles an ecological momentary intervention (EMI), which delivers real-time information to patients during their everyday lives. A total of 13 other EMI programs designed to address anxiety symptoms in patients exist currently [30]. Similar to Cope Notes, these programs attempt to improve symptoms through mobile technology–based psychoeducation, but only 2 deliver similar intervention-based SMS text messages [31].

Cope Notes differs from other SMS text messaging–based behavioral interventions, such as Text4Mood, because of the founder’s personal lived experience with mental illness which enabled him to create an authentic program that resonates with subscribers in search of additional support [32]. Cope Notes aims to use evidence-based approaches, including positive psychology, stigma reduction messaging, and cognitive restructuring, to impact users’ mental well-being, as well as encourage help seeking skills in potential users through peer support. By specifically applying SMS text messaging to these concepts, Cope Notes stands as a unique addition to existing intervention programs.

### This Study

Apart from certain elements seen in Text4Mood, such as text messages based on principles of cognitive behavioral therapy to target mood and anxiety and promotion of mental well-being [33], no other behavioral health mHealth interventions discussed in the existing literature incorporate SMS text messaging to promote positive mental health self-management using a combination of cognitive restructuring, positive psychology, and stigma reduction. The purpose of this research study is to understand user experiences with Cope Notes as an SMS text messaging–based, mental health, self-management intervention. By understanding the experiences of Cope Notes subscribers, this mixed methods study [9] will facilitate the future application of these findings to other text message–based interventions through a phenomenological lens and advance findings surrounding SMS text messaging–based mHealth. We also aim to identify the current acceptability and usability of Cope Notes to improve similar mHealth services and close the gap in literature as it relates to stigma and how personality influences mHealth use and outcomes in mental wellness. Although in this study we do not investigate the application and use of ubiquitous sensing as it applies to SMS text messaging–based mHealth, we aim to uncover user perceptions of ubiquitous sensing in this space. We view ubiquitous sensing as a potential future innovation of the Cope Notes intervention and have used this research as an opportunity to assess the acceptability of this potential application in future research and development activities. Specific research questions we sought to answer with this study include the following:

1. How do Cope Notes users perceive the service as it relates to stigma, impact of the intervention, and perceived usefulness?
2. How do Cope Notes users rate the Cope Notes service and text messaging along various dimensions of acceptability?
3. What is the relationship between Cope Notes message ratings, user personality, and coping strategies?
4. What are user perspectives of ubiquitous sensing technologies, including integration of ubiquitous sensing for the improvement in the timeliness of the intervention and quality of tailored content?

## Methods

### Ethics Approval

This study was approved by the University of South Florida’s (USF) Institutional Review Board (Pro00040410).

### Study Design

To answer our research questions which guided this mixed methods study, we partnered with the USF Morsani College of Medicine (MCOM) and the chief executive officer of Cope Notes to facilitate convenience sampling strategies. We aimed to include 15 participants in the study as this number was deemed large enough to expect to achieve saturation in the themes emerging from the interviews [34]. We collected qualitative and quantitative data separately, and the quantitative component consisting of inventories collected after qualitative interviews was assessed. Qualitative data collection included (1) a demographic questionnaire and (2) qualitative interviews to inform the first and fourth research questions in the aforementioned list. The first quantitative measure evaluated a (3) user experience survey and addresses our second research question. The remaining quantitative data collected, that is, (4) a personality inventory and (5) coping assessment, provided a means to evaluate our third research question in the aforementioned list. Following data collection, we analyzed the qualitative and quantitative data using ATLAS.ti and SPSS (version 26.0; IBM Corp), respectively. The quantitative aspects of the study are used to inform and enhance the themes and patterns that emerged in the qualitative data and SPSS.

### Recruitment and Sample

Inclusion criteria required participants to be aged ≥18 years and subscribed to Cope Notes for at least 30 days. Exclusion criteria included not being able to read or speak English. Partnership with the chief executive officer of Cope Notes and USF MCOM guided our recruitment strategies. First, the partnership with Cope Notes allowed a text message to be sent out to subscribers asking whether they would be interested in participating in the study. The USF MCOM partnership provided medical students access to a 30-day Cope Notes subscription as a wellness resource during a time of high stress—the study period before the students’ board exam. These gift subscriptions helped us gain additional participants. Recruitment strategies for medical students included sending out emails requesting participation in the study and verbally sharing information about the study in person at lectures. Volunteering subscribers were contacted via email to schedule in-person, Skype, or telephone interviews depending on their preference and availability. All participants provided informed consent before the commencement of the study and were compensated a digital gift card worth US $15 for their participation. A total of 14 participants were included in this study; 9 (64%) were recruited through the Cope Notes partnership, whereas 5 (36%) were recruited through the MCOM. All data for this study were collected from June 2019 to December 2019.

### Qualitative Study Design

Qualitative interviews were conducted to learn more about user experiences with the Cope Notes service, unearth perceptions on privacy and acceptance related to ubiquitous sensing, and gather thoughts pertaining to mental health stigma. Coupled with the interviews, the demographic questionnaire in Multimedia Appendix 1 helped categorize experiences shared by participants—providing context to the outcomes and trends seen in the interviews. Live interviews were conducted using a semistructured interview guide (Textbox 1) and were audio recorded. The interviews lasted no more than an hour and on average lasted approximately 35 minutes, including completion of the demographics survey, and were transcribed verbatim using a secure web-based transcription service.

**Comprehensive list of interview questions from the qualitative interview guide.**

**Interview questions**

1. What did you like or dislike about Cope Notes?
2. If you responded to any of the texts, describe your experience.
3. Would you recommend any changes or additional functionality to Cope Notes?
4. How do you feel about the delivery strategy of randomly timed messages?
5. Would you recommend any changes to the current delivery strategy?
6. We are considering creating a companion app that you could run in the background on your phone that would gather data using your phone’s sensors. What do you think about this?
7. Here are some examples of the data we would collect from smartphone sensors and how it would allow us to predict levels of stress, anxiety, or depression. Do you have any reactions to this?
8. If Cope Notes has been helpful for you, in what areas of your life has it been helpful?
9. Can you recall the most and least helpful texts you received form Cope Notes; why were these the most or least helpful?
10. How do you feel about the cultural sensitivity of Cope Notes?
11. How does knowing the founder of Cope Notes has lived experience with mental illness change your experience or impression of Cope Notes?
12. How do you think that Cope Notes might affect stigma surrounding mental illness?

### Quantitative Study Design

#### Quantitative Components and Measures

**Figure 1:**
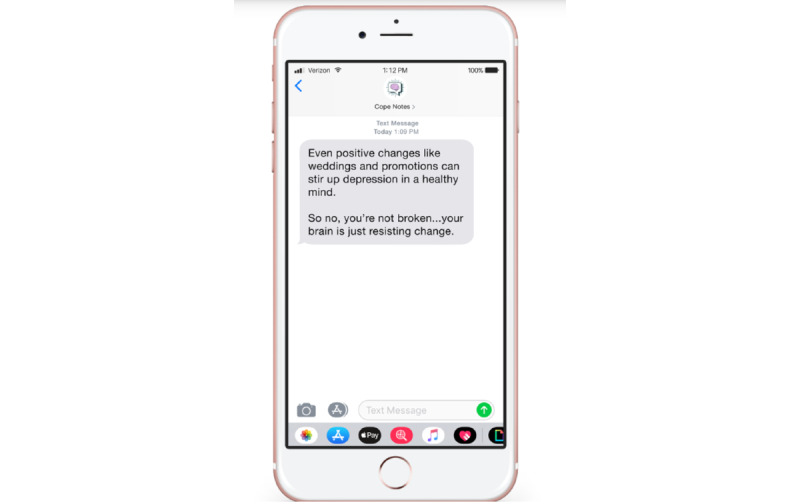
Example of an SMS text message that subscribers receive from the Cope Notes service encouraging cognitive reframing.

**Figure 2:**
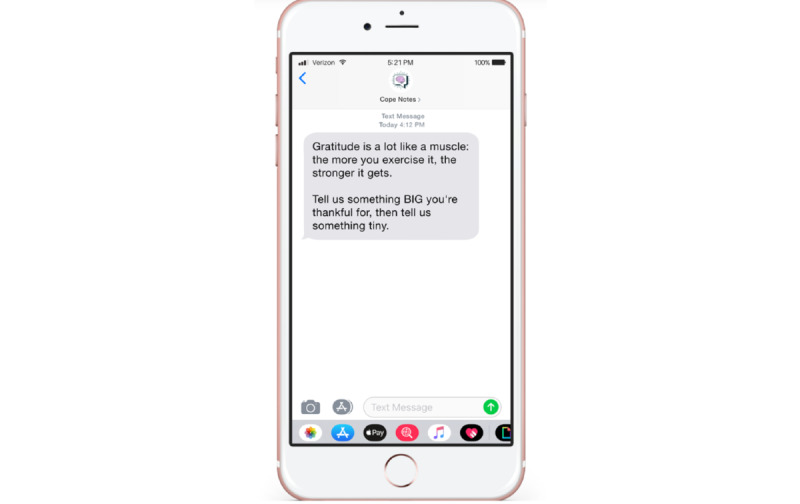
Example of an SMS text message that subscribers receive from the Cope Notes service encouraging reflection.

The quantitative component of this study consisted of a user experience survey, a personality inventory, and an inventory of coping strategies. The user experience survey served to directly measure how impactful certain elements of Cope Notes are perceived by participants. The personality inventory and coping assessment was used to extract possible connections between interactions and perceptions of the Cope Notes service and specific characteristics regarding the participants who engaged with the service. In this way, the quantitative aspects of the study are used to enhance the analysis of patterns that are present in our qualitative results. All quantitative measures were delivered securely and electronically through the Qualtrics survey software, and participants completed the surveys independently without the support of a researcher. Quantitative measures are presented in Multimedia Appendix 2. Examples of Cope Notes messages received by participants are presented in Figures 1 and 2.

#### User Experiences Survey

The participants were asked to rate Cope Notes on several dimensions approximating the acceptability of the program, including (1) relevance to the user’s own life, (2) timing of delivery, (3) how much reflection the texts elicited, (4) how much positive or negative emotion the texts elicited, (5) the user’s understanding of the text’s sentiment, (6) how much the texts promoted cognitive restructuring and coping, and (7) how much the texts inspired users to share the message with others. A total of 6 items were created in consultation with the chief executive officer of Cope Notes. The internal consistency of the 7 items used in this study was Cronbach *α*=.874, demonstrating sufficient reliability [35]. Items on the survey were answered on a 7-point Likert scale (7=strongly agree). The participants were instructed to rate their level of agreement with a series of statements regarding the content (corresponding to the domains listed) of Cope Notes messages received over the past 30 days. An example item from this scale was, *Cope Notes messages were relevant to my life as a whole, regardless of when they were received.* This measure is available in Multimedia Appendix 2. Because of the exploratory nature of this preliminary investigation of Cope Notes acceptability, we did not use a psychometrically validated instrument to assess user experiences; however, existing research with a similar goal of assessing the acceptability of a TMI adopted a similar methodological approach [24,33].

#### Personality Inventory

Personality was measured using a short 10-item version of the Big Five personality inventory, the Big Five Inventory (BFI)-10 [36]. This scale was evaluated against the BFI-44 and the Neuroticism, Extraversion, and Openness to experience Personality Inventory-Revised for reliability and validity. BFI-10 scales retain significant validity and reliability, making it a reliable measure for use in this study. The BFI-10 is a shortened version of the original BFI with 44 items. The BFI-10 was created to decrease the required assessment time carried out by researchers. This measure includes 2 items for each of the 5 dimensions of the Big Five—openness, conscientiousness, extraversion, agreeableness, and neuroticism.

#### Coping Assessment

Coping was measured using the Brief Coping Orientation to Problems Experienced (COPE) inventory [37], which has also demonstrated good reliability and validity [38]. Brief COPE is an abbreviated version of the original COPE Inventory, a questionnaire that assesses a broad range of coping strategies. Brief COPE contains 14 two-item subscales rather than the original 60-item inventory. The reduced multidimensional measures of coping in response to stressors are analyzed separately as follows: (1) self-distraction, (2) active coping, (3) denial, (4) substance use, (5) use of emotional support, (6) use of instrumental support, (7) behavioral disengagement, (8) venting, (9) positive reframing, (10) planning, (11) humor, (12) acceptance, (13) religion, and (14) self-blame [39].

## Results

### Sample Characteristics

Of the 14 participants, 10 (71%) identified as White, 2 (14%) identified as Middle Eastern or Arab American, 2 (14%) identified as Asian American or Asian, 1 (7%) identified as Black or African American, and 1 (7%) identified as Pacific Islander. Half of our sample (7/14, 50%) identified as male, and 43% (6/14) identified as female. It was noted that 71% (10/14) of the individuals had consulted a mental health professional (psychologist, psychiatrist, etc) for challenges regarding their mental well-being (eg, emotional, behavioral, and cognitive), and 64% (9/14) had a diagnosis of a mental illness. Of that 64% (9/14), participants specified being diagnosed with clinical depression, anxiety, bipolar depression, and attention deficit disorder. In all, 29% (4/14) of the participants reported not receiving a diagnosis of a mental illness. Table 1 provides an overview of the participant characteristics, which were obtained through the demographic survey. On average, participants had been active Cope Notes subscribers for 190 (SD 194) days. The minimum requirement for participation in the study was at least a 30-day subscription, whereas the earliest subscriber had a 700-day long subscription at the time of their interview. The demographic survey completed by participants is available in Multimedia Appendix 1.

**Table 1:** Participant characteristics collected from the demographic survey (N=14).

Characteristics			Participants, n (%)
**Age (years)**			
	18-19	1 (7)	
	20-29	5 (36)	
	30-39	3 (21)	
	40-49	1 (7)	
	50-59	2 (14)	
	Undisclosed	1 (7)	
	Missing	1 (7)	
**Gender**			
	Female	6 (43)	
	Male	7 (50)	
	Missing	1 (7)	
**Race**			
	African American or Black	1 (7)	
	Asian or Asian American	2 (14)	
	White	10 (71)	
	Undisclosed	1 (7)	
	Missing	1 (7)	
**Diagnosis of mental illness**			
	Yes	9 (64)	
	Anxiety	0 (0)	
	Depression	4 (29)	
	Anxiety and depression	5 (36)	
	No	4 (29)	
	Missing	1 (7)	

### Qualitative Findings

#### Overview

The qualitative interview data were analyzed by 2 independent coders (SLK and JL) using structured coding methods following a narrative analysis approach within the ATLAS.ti program [40]. The study team derived overarching themes of interest driven by our research questions (ie, Likes or Perceived Benefits) and minor themes that emerged during the coding process (ie, customized delivery strategy). Interrater reliability, the percentage agreement between the 2 coders (SLK and JL) calculated using Cohen *κ* in SPSS, was 61.8%, showing substantial rater agreement [41]. Table 2 displays the 52 minor codes and how they are organized into 7 overarching themes: Likes or Perceived Benefits, Dislikes or Limitations, Suggested Changes, Stigma or Help Seeking, Perceptions of Ubiquitous Sensing, Cultural Sensitivity, and Alternative mHealth Resources (see Multimedia Appendix 3 for definitions of the 52 minor codes). Disagreements were resolved through discussion and coming to a consensus as a team.

**Table 2:** Themes, code frequency, and number of participants reporting each code. Most frequent codes per theme are provided below. See Multimedia Appendix 3 for definitions of all 52 minor codes (N=14).

Theme and code		Code frequency	Participants, n (%)
**Likes or perceived benefits^a^**			
	1	122	14 (100)
	2	66	12 (86)
	3	50	14 (100)
	4	24	9 (64)
	5	38	13 (93)
	6	68	14 (100)
	7	39	11 (79)
	8	24	11 (79)
	9	17	8 (57)
	10	4	4 (29)
	11	2	2 (14)
**Dislikes or limitations^b^**			
	12	13	5 (36)
	13	12	3 (21)
	14	23	5 (36)
	15	21	6 (43)
	16	11	7 (50)
	17	4	2 (14)
	18	2	1 (7)
**Suggested changes^c^**			
	19	9	4 (29)
	20	14	9 (64)
	21	25	4 (79)
	22	8	7 (50)
	23	6	3 (29)
	24	1	1 (7)
	25	3	3 (21)
**Stigma or help seeking^d^**			
	26	10	5 (36)
	27	11	6 (43)
	28	39	12 (86)
	29	46	12 (86)
	30	38	12 (86)
	31	43	12 (86)
	32	9	4 (29)
**Alternative mHealth^e^**			
	33	14	5 (36)
	34	2	1 (7)
	35	22	7 (50)
	36	9	3 (21)
	37	1	1 (7)
**Perceptions of ubiquitous sensing^f^**			
	38	33	10 (71)
	39	18	9 (64)
	40	13	8 (57)
	41	11	5 (36)
	42	10	7 (50)
	43	9	6 (43)
	44	14	7 (50)
	45	16	9 (64)
	46	12	6 (43)
	47	4	2 (14)
	48	8	4 (29)
	49	3	2 (14)
**Cultural sensitivity^g^**			
	50	8	6 (43)
	51	11	9 (64)
	52	3	2 (14)

^a^Most frequent code: positive impact.

^b^Most frequent code: lack of impact.

^c^Most frequent code: customized message content.

^d^Most frequent code: stigma reduction.

^e^Most frequent code: other mHealth or therapy.

^f^Most frequent code: positive reaction.

^g^Most frequent code: neutral cultural impact.

#### Theme 1: Likes or Perceived Benefits

When asked about Cope Notes, users generally found Cope Notes to be helpful and supportive:

Cope Notes to me is exactly like somebody saying “hello” to somebody. “How are you doing?”

Participant 6

Cope Notes would come in and really help me focus on what I could control, and what I couldn’t, and what was important.

Participant 7

All participants voiced a preference for convenience, including passive, low effort, and low time commitment resources that were simple to use:

I think it’s a quick tool and it doesn’t really take that much effort. That’s what I like about it.

Participant 3

...text messages, which is about as easy as it gets.

Participant 1

Of the 14 users, 12 (86%) noted that Cope Notes encouraged positive reframing of their mental wellness, and 13 (93%) stated that they liked the random message timing:

I like that it comes at different times of day...[the messages] have been helpful to me, good reminders.

Participant 9

It’s just a nice little way to recenter my thoughts in the middle of the day...I like that they come in randomly, not at the same time every day.

Participant 7

All users (14/14, 100%) said that they liked the variety of topics, as well as the subject depth, that Cope Notes delivered to them. Some users (4/14, 29%) felt the messages even triggered awareness, self-reflection, and mindfulness when it came to their everyday interactions:

Cope Notes is great because it gives you something to think about and to reflect on.

Participant 3

#### Theme 2: Dislikes or Limitations

A common dislike was the invariance of message length:

...message length was always...standardized.

Participant 1

Of the 14 users, 6 (43%) found that the tone of some of the texts seemed more off-putting rather than encouraging, and in some cases, not sufficiently engaging:

Some [text messages] are instructional which to me felt kind of weird.

Participant 2

It was just quotes. I saw other people got more interactive [messages].

Participant 4

In all, 50% (7/14) of the participants also noted that the timing of some messages came too late or too early in the day to be viewed as helpful:

It would come so late that I was already asleep.

Participant 4

In total, 14% (2/14) of participants also stated that Cope Notes was “too expensive” as follows:

I’m not sure I would pay $10 a month for it...when life was crazy, I would pay a little bit more.

Participant 3

In addition, a user experienced multiple text threads instead of a consistent conversation thread of daily messages and found that to be cumbersome:

For me it was always from a different number, so my inbox was [a] whole bunch of Cope Notes.

Participant 4

#### Theme 3: Suggested Changes

The most common suggested change, recommended by 64% (9/14) of users, was to include more interactive media, such as inspirational wallpapers or images:

I’m more of a visual person...that would kind of enrich the experience.

Participant 2

Users also suggested variability in the length of text messages to make it less robotic. Similarly, another popular suggestion made by 29% (4/14) of participants was the ability for a user to choose the time frame of delivery, as well as the type of content received on a day-to-day basis:

I prefer to be able to set it at a time that I feel like I need it most...I’m like, okay, it’s 4:15 [and] I haven’t received my message yet. But I know that it is coming somewhere along in the afternoon.

Participant 8

A user suggested the ability to bookmark text messages that they wanted to remember, whereas 50% (7/14) of the users requested an increase in 2-way interactions:

Ooh [if] you can favorite things...have a favorite quote to come back [to].

Participant 3

[I’d prefer] a personalized [response] for sure, if it was automated, I probably wouldn’t respond.

Participant 11

#### Theme 4: Stigma or Help Seeking

Although the consensus reported by 86% (12/14) of users was that Cope Notes could assist in stigma reduction, participants raised 2 alternative opinions worth noting. First, some (5/14, 36%) participants stated that upfront knowledge of the Cope Notes founder’s lived experience with mental illness could deter those who are label avoidant. Label avoidance occurs when someone avoids people or places that might prime a stigmatizing label such as mental illness. In other words, people may shy away from receiving mental health services (continuing or initiated services) due to fears of being associated with the negative stigma surrounding mental illness. As such, using the founder’s public background of lived experience as a selling point of this service may discourage others from using Cope Notes to avoid stigma.

Second, 86% (12/14) of participants acknowledged that Cope Notes assisted them with reflecting on shared previous experiences and perceptions of stigma that they could tie to preferred styles of peer support:

[Cope Notes] doesn’t allow the opportunity for stigma to kind of present itself, because it’s applicable across the board.

Participant 7

I think it’s really totally helping people remove the stigma of mental health issues. It’s normalizing that most of the population have issues with mental illness.

Participant 12

Participants also stated that Cope Notes may encourage some users to find more professional forms of mental health treatment, although some were not sure which of their peers would intentionally seek professional mental health services:

I could see people picking something more light, like Cope Notes, before trying...medical attention type coping mechanisms.

Participant 2

I don’t trust counselors...[Cope Notes] is kind of like a nice little...a form of counseling.

Participant 1

#### Theme 5: Perceptions of Ubiquitous Sensing

We asked participants to consider the use of a companion app as a complement to the traditional Cope Notes SMS text message service. The app’s functionality, that of which reflects a ubiquitous sensing-like paradigm, was explained to participants in plain language as follows. The app would use the embedded sensors (participants were also provided with this information during their interview; Textbox 2) in the user’s smartphone to gather information about the user’s surroundings and how the user interacts with their device. These data would then be used as input to a machine learning algorithm trained to identify distinct patterns in the user’s behavior to predict instances where the user might be upset, stressed, or expressing otherwise concerning behavior. Such an app would run in the background on the user’s smartphone, and based on the prediction from the algorithm, trigger an SMS text message intervention to be sent to the user, providing timely support when users need it the most.

**Sensor descriptions presented to users accompanying the interview questions on a Cope Notes companion app.**

**Sensor descriptions**

- Your smartphone’s hardware consists of a variety of sensors that can gather information from your environment.
- These sensors work together in a continuous and transparent fashion to process that information into something meaningful for you.
- For example, if you use an app for tracking your daily runs, the accelerometer sensor is used for tracking that movement. Researchers have been studying sensor data for many reasons; one of these reasons is to predict abnormal activity (eg, you skipped one of your morning runs), which may correlate with certain moods such as stress or anxiety. The items below are some examples of sensor data that might be collected to predict your stress and anxiety level.
- What are your reactions to these items and this form of data collection?
- Calling and text messaging activity can include data such as the partial phone number of the receiver, time of the call, and length of the call. Research has shown that call and text messaging patterns may be associated with anxiety.
- The screen’s status (ie, on or off) and the light sensor (which measures the light source around you) can be used to approximate your sleeping patterns, a known indicator of stress.
- When surrounded by other devices, your smartphone may be able to detect them via Bluetooth technology. Thus, the number of devices detected by your smartphone may provide an approximation of your social context.
- GPS and Wi-Fi sensor information are excellent for approximating location. Studies have shown that location patterns provide valuable insight regarding mood.
- The accelerometer and gyroscope sensors detect speed and rotation and are thus commonly used for recognizing physical activities. When unusual, your activities may be an indication of a potential problem.
- The microphone’s gathering of sound information can be processed at a high level for recognizing various sound sources, like wind or motorized transport, to be used for approximating your environment.
- Similar to call logs, app logs track your use of mobile apps and other statistics such as when an app is installed or opened. This information can provide insight into your interests and overall use of the device.

Most participants were neutral (8/14, 57%) or responded positively (10/14, 71%) to the idea of the app providing more timely interventions according to need:

If someone’s stressed out, I think a great thing is to have someone to just give them...[a] nugget of wisdom.

Participant 1

I think that would just be a great enhancement, as a resource!

Participant 8

In all, 50% (7/14) of the users were concerned that other users may be concerned about privacy, whereas 64% (9/14) of participants had concerns about the collection of data themselves:

It’s a little big brother-y, I think is what people think.

Participant 7

I think this also might be perceived by a lot of people as an intrusion of their privacy.

Participant 10

Many participants (6/14, 43%) mentioned specific sensors that they had a negative reaction to (ie, call logs and microphone). As a remedy, participants suggested allowing users to select which sensors the app could access:

Let people pick which data to allow the app to use.

Participant 2

Meanwhile, only 14% (2/14) of participants were opposed to using the sensors owing to their personal privacy concerns:

I actually would not be interested in that...Yeah, it’s a privacy issue.

Participant 13

I definitely struggle with like the microphone gathering information, listening...I don’t think I’d leave that up to my phone, my phone data to gauge [my behavioral patterns].

Participant 14

Furthermore, 43% (6/14) of participants were unsure of the abilities of the app or underlying machine learning algorithm to predict times when a user may be stressed or needs support:

It’d be interesting to see how well it worked and how accurate it was.

Participant 9

#### Theme 6: Cultural Sensitivity

When asked about cultural sensitivity, participants responded in a generally neutral (9/14, 64%) to positive (6/14, 43%) manner:

I haven’t ever come across anything that seemed like it was directed or misdirected.

Participant 7

Participants did note that most statements made in the messages received referenced Western culture related references:

References to a famous person...someone in another country may not [know them].

Participant 1

#### Theme 7: Alternative mHealth Resources

Another theme that was recognized was the comparison of Cope Notes to other mobile mental wellness resources. In all, 36% (5/14) of participants compared this text message program to the meditation mobile app Headspace or the gratitude journal mobile app Three Good Things:

Three Good Things is very unique and individual, right? And it’s also very active, so you...unlike Cope Notes, you can’t ignore it, it requires you to put in something, it’s not just a message that comes and you read it.

Participant 10

A participant also compared it to Autonomous Sensory Meridian Response videos, which are web-based videos that include sensation inducing audio triggers. Cope Notes was also compared with Early Alert, a text message mental wellness check program designed for professional health students. However, users distinguished that these programs were for specific parts of mental health whereas Cope Notes was more encompassing:

I think it’s easier. In general, to get to more people with Cope Notes than you can with Not A Therapist.

Participant 15

### Quantitative Findings

We conducted exploratory analyses using descriptive statistics and correlations. Specifically, the quantitative survey data were analyzed through descriptive statistics using SPSS. We were interested in the mean user ratings of Cope Notes’ acceptability as well as how personality and coping strategies might relate to user ratings. Table 3 provides the means and SDs of participant ratings of Cope Notes acceptability along 10 dimensions. The sample size for each item varies as participants were given the option to respond that the question was not applicable. In addition, not all participants completed the quantitative survey. Responses to the survey were obtained from 12 of the 14 (86%) participants who provided qualitative interviews. Higher scores (scores could range from 0=not applicable to 7=strongly agree) represent more agreement with each of the acceptability items.

**Table 3:** Means and SDs of acceptability rating items of the Cope Notes service (N=14).

Acceptability rating items	n (%)	Mean (SD)
1. Cope Notes messages were relevant to my life as a whole, regardless of when they were received.	12	6.25 (1.06)
2. Cope Notes messages came at a relevant time in my life.	12	6.08 (1.38)
3. Cope Notes messages were not relevant to my life at all.	5	2.20 (0.45)
4. I think of Cope Notes messages often, and remembering them helps me face new situations.	11	5.73 (1.42)
5. Cope Notes messages provoked a positive feeling.	12	6.08 (0.90)
6. Cope Notes messages provoked a negative feeling.	4	2.00 (0.00)
7. I fully understood the sentiment behind Cope Notes messages.	12	6.08 (1.16)
8. Cope Note messages helped me view myself or my situation differently.	12	6.08 (1.08)
9. Cope Notes messages helped me deal with or relieve pressure or stress.	12	6.00 (1.47)
10. I have shared Cope Notes messages with others or posted them on a social networking site.	10	6.00 (1.25)

Exploratory analyses of the relationship between acceptability ratings and personality factors as measured by the BFI-10 revealed several significant correlations. Spearman correlations indicated a statistically significant positive relationship between seeing oneself as someone who is generally trusting and acceptability items 4 (*r*_s(9)=_0.71, *P*=.01), 5 (*r*_s(10)_=0.75, *P*=.005), 7 (*r*_s(10)_=0.82, *P*=.001), and 10 (*r*_s(8)_=0.71, *P*=.02). There was also a significant positive correlation between perceptions of oneself as someone who does a thorough job and acceptability item 8 (*r*_s(10)_=0.80, *P*=.01).

Examining relationships between Brief COPE items and acceptability ratings revealed a statistically significant negative correlation between receiving emotional support from others and acceptability item 1 (*r*_s(8)_=−0.80, *P*=.006) and 9 (*r*_s(8)_=−0.79, *P*=.006). The active coping strategy of taking action to make things better was significantly positively related to acceptability item 8 (*r*_s(8)_=0.72, *P*=.02). Endorsement of the coping strategy of saying things to let one’s unpleasant feelings escape was significantly negatively related to acceptability item 5 (*r*_s(8)_=−0.66, *P*=.04) and 7 (*r*_s(8)_=−0.70, *P*=.03). Participants who strongly endorsed the coping strategy of doing something to think less about their problems (eg, going to movies, watching television, reading, daydreaming, sleeping, or shopping) were significantly less likely to strongly endorse acceptability items 5 (*r*_s(8)_=−0.74, *P*=.01) and 7 (*r*_s(8)_=−0.75, *P*=.01). Participants strongly endorsing coping by accepting the reality that an event has happened also strongly endorsed acceptability items 5 (*r*_s(8)_=0.69, *P*=.03), 7 (*r*_s(8)_=0.86, *P*=.001), 9 (*r*_s(8)_=0.67, *P*=.03), and 10 (*r*_s(6)_=0.84, *P*=.009). Finally, endorsement of using the coping strategy of expressing negative feelings was significantly positively related to acceptability item 8 (*r*_s(8)_=0.67, *P*=.03).

## Discussion

### Principal Findings

In general, Cope Notes users found Cope Notes to be helpful and supportive. Specifically, users spoke about Cope Notes functioning to help them refocus on aspects of their lives over which they had control. Users liked that Cope Notes is a low-effort tool and that it is easy to use and saw Cope Notes helpful for managing stigma and normalizing mental illness. Users also framed Cope Notes as a low-intensity intervention [42] that may lead to seeking professional mental health care later. Users reported that Cope Notes helped with reframing their mental wellness and liked the random timing of the messages. These positive reactions to Cope Notes are confirmed by the high levels of acceptability endorsed through the quantitative survey asking users to rate the messages on the 10 dimensions of acceptability.

On the other hand, Cope Notes users disliked the standard message length and occasionally disliked the tone of a text message. Some (3/14, 21%) users did not like the early morning or late-night delivery of a text. Some (2/14, 14%) participants also felt that the subscription cost was too high. Changes suggested by users included requests for more media-based and engaging texts, an option to select the time of message delivery, and a more personalized experience. Users generally had a positive or neutral response to the use of ubiquitous sensing, which might aid in bringing about such changes. No specific concerns were expressed about the cultural sensitivity of Cope Notes, though it was noted that Cope Notes seems to be targeted toward Western cultures. We also found that users compared Cope Notes to other mHealth interventions; however, they viewed Cope Notes as targeting mental health more broadly than other products. The goal of our qualitative inquiry was not to generalize these findings but instead to gain understanding of the experiences of users of this SMS text messaging mHealth self-management resource.

There were several personality traits that were significantly related to the acceptability ratings of Cope Notes. Seeing oneself as generally trusting and acceptable was positively related to remembering the Cope Notes messages and using them to face new situations, positive feelings provoked by the Cope Notes texts, fully understanding the sentiment behind the texts, and sharing the Cope Notes messages with others or on social media sites. These relationships seem to reflect the notion that those who trust others may be more likely to place trust in the content of the texts, thereby experiencing a positive reaction to the texts and a desire to share them with others [43,44]. Perceptions of oneself as doing a thorough job was positively related to responding that the Cope Notes texts helped the user to view themselves and their situation differently. This too seems reasonable as those who may have thoroughness as a personality trait may be more likely to process and use the information contained in the text messages [45]. These findings regarding personality provide insight into the types of users who may be more likely to find Cope Notes, or similar SMS text messaging–based interventions, as an acceptable tool for coping, and may also inform the modification of the intervention to appeal to a broader range of personality types.

The coping strategy of receiving emotional support from others, as measured by the Brief COPE, was negatively related to viewing Cope Notes messages as relevant to one’s life and using the messages to help deal with pressure or stress. This may suggest that Cope Notes would be most acceptable to those who lack emotional support from others in their immediate environment. Coping styles of taking action to make things better was positively related to using the messages to help change one’s views of themselves or their situation. This was a logical finding as both are active forms of coping, also referred to in the existing literature as problem focused coping [46]. There was a negative relationship between endorsing positive feelings provoked by Cope Notes and fully understanding the sentiment of the text and the coping strategy of saying things to let one’s negative feelings escape. Perhaps those users who vent more are less likely to experience a positive response to a nonverbal text message and therefore may be less likely to exert effort to understand the message. Those participants who endorsed the coping strategy of doing things to distract themselves from their problems were also less likely to experience positive feelings from Cope Notes messages or understand the message’s sentiment. This finding might reflect the idea that the passive receipt of a text is less likely to emotionally impact those who prefer such active coping strategies and therefore less likely to inspire an effort to understand message content. Participants who endorsed coping by accepting the reality of an event also endorsed positive feelings evoked by Cope Notes, understood message sentiment, used Cope Notes to relieve pressure or stress, and shared the messages with others. Many of the Cope Notes texts are rooted in positive psychology strategies such as mindfulness and acceptance and therefore users who tend to cope in this way may be particularly likely to have a positive experience with Cope Notes. Taken together, these findings may inform targeting of the Cope Notes customer segment as well as modifications of the Cope Notes intervention or content of texts to respond to a broader range of coping styles. Despite the scarcity of similar investigations to compare findings, in summary, we found that those with active coping tendencies, or those who are more likely to receive outside emotional support are less likely to feel positively impacted by Cope Notes. However, participants who are more likely to cope through nonverbal approaches are more likely to embrace Cope Notes. We continuously received feedback from participants who were more concerned about internet safety and privacy regarding the possibility of a companion app from a group think perspective. Individually, participants were not as concerned about privacy and security as they believed other possible users to be.

### Comparison With Previous Work

Text4Mood is a similar SMS text messaging–based program that sends supportive texts each day to users located in North America who text the word *mood* to a specific number. This program is designated for those who are currently on a wait list to receive services or have *difficulty accessing service due to geographic barriers* [24]. Text4Mood can also provide psychological support for those currently enrolled in individual or group counseling. For US $5.40, users can access this service for 6 months at no additional charge.

Text4Mood differs from Cope Notes as it is marketed for users who are in the process of receiving treatment directly from a provider, whereas Cope Notes does not require users to pursue direct care. The geographic boundaries and price points also differ; Text4Mood is only available to users in North America, whereas Cope Notes does not have any geographic limitations and the subscription ranges from US $6.99 to US $9.99 per month (depending on the type of subscription purchased). Users of Cope Notes and Text4Mood both reported positive benefits to their overall mental well-being after extended use of both services.

Cope Notes is also similar to EMIs, as previously referenced in the Introduction section. Heron and Smyth [30] identified 27 EMIs for different health conditions, 6 of which focused on addressing symptoms of anxiety. None of these interventions, including the 6, used SMS text messaging [30]. A more recent meta-analysis of EMI interventions meant to augment mental health and positive psychological well-being, which included some SMS text messaging–based interventions, found that EMIs were effective (medium effect sizes) for improving anxiety, depression, perceived stress, acceptance, relaxation, and quality of life [47]. However, none of the illness self-management SMS text messaging–based EMIs reviewed in the literature to date attempt to integrate cognitive restructuring, positive psychology, and stigma reduction messaging, and none involve a component of peer support, making Cope Notes an innovative addition to the existing menu of available EMIs for mental illness and its evaluation concerning effectiveness and acceptability, a novel and timely addition to research literature in mHealth.

### Limitations

The small sample size for our quantitative survey limits our ability to gain an understanding of the relationship between personality and coping styles and the acceptability of the Cope Notes program on a broader scale. Despite our efforts to recruit a large, heterogenous sample, ultimately our relationship with the USF MCOM for recruitment yielded a significant portion of research participants, which could have implications regarding the generalizability of this work. However, we also aimed to reach a more representative population of the Cope Notes users via an SMS text message blast sent to subscribers. It is also possible that the demographic information present in our sample is largely representative of Cope Notes subscribers. Regarding the sample size, we continued to recruit participants until we noticed a clear pattern of qualitative data providing repetitious information. After reviewing the data from the first 14 participants, we then determined we had reached saturation through repeated emerging themes and ended the recruitment stage.

We did detect some statistically significant and potentially meaningful relationships that could be used to target the Cope Notes audience segment and modify the program or message content; however, additional research with a larger sample size is called for to gain a more accurate picture of those who might be more likely to benefit from Cope Notes and SMS text messaging–based interventions in general.

### Conclusions

Affordability, limited numbers of mental health providers, stigma, and other factors limiting accessibility to or willingness to seek mental health services are well known. These barriers to care are exacerbated by the effects of the COVID-19 pandemic, further increasing the demand for accessible mental health support [48,49]. mHealth interventions are one way to enhance the accessibility of mental health services, although all forms of mHealth interventions, such as SMS text messaging–based interventions, are understudied [50]. Understanding the effectiveness of mHealth resources, such as SMS text messaging–based intervention Cope Notes, for improving and facilitating mental health self-management services is more important now than ever before.

The presented mixed methods study found subscribers of Cope Notes to appreciate the service for refocusing and reframing their mental wellness, with noted statistically significant correlations between specific personality traits and acceptability of the SMS text messaging–based service as evaluated via qualitative interviews, coping assessment, personality inventory, and a user experiences survey. However, we also found that some users preferred a more personalized experience with Cope Notes, with a potential for a companion app equipped to continuously monitor user behavior to identify moments of distress as a viable solution. Despite touching on ubiquitous sensing in this way, investigating an actual implementation of ubiquitous sensing was not an objective of this study. Findings of this study regarding participant personality and coping styles and Cope Notes ratings indicate potential avenues for future research on coping styles and personality as mediators of acceptability of text-based mental health interventions. It is important to note that Cope Notes is not a replacement for professional mental health treatment but instead a supplement for professional forms of treatment. Through these findings, other researchers can gain more understanding to how SMS text messaging may be received by others, even outside of behavioral interventions. Future research will include a more refined focus on the possibility of using ubiquitous sensing in text message–based interventions for mHealth purposes.

## Appendix

Multimedia Appendix 1 Demographic survey completed by the study participants.

Multimedia Appendix 2 User experience survey completed by the participants, including the Brief Coping Orientation to Problems Experienced Inventory and Big Five Inventory-10.

Multimedia Appendix 3 All minor codes, their definitions, and their corresponding themes.

## Abbreviations

BFI: Big Five Inventory
COPE: Coping Orientation to Problems Experienced
EMI: ecological momentary intervention
MCOM: Morsani College of Medicine
mHealth: mobile health
TMI: text message intervention
USF: University of South Florida
