# Supplementary material for: Characterizing User Experiences With an SMS Text Messaging–Based mHealth Intervention: Mixed Methods Study
Source: JMIR Form Res. 2022 May 3;6(5):e35699. doi: 10.2196/35699 (PMC9115655; doi:10.2196/35699)
Supplement: Multimedia Appendix 1 [file formative_v6i5e35699_app1.pdf]

This is a Multimedia Appendix to a full manuscript published in the JMIR Form Res. For full copyright and citation information see https:// doi.org/10.2196/35699.

Cope Notes Participant Demographics Questionnaire

1. Please provide the email address used to sign up for the Cope Notes study group.
2. Please provide your age in years:
3. What is your gender?
    a. Cisgender Male
    b. Cisgender Female
    c. Transgender
    d. Other (Please specify):
    e. Prefer not to answer.
4. What is your race/ethnicity? (Check all that apply)
    a. White / Caucasian
    b. African American / Black
    c. American Indian / Alaskan Native
    d. Arab / Middle Eastern / Arab American
    e. Asian / Asian American
    f. Pacific Islander
    g. Other (Please specify):
    h. Prefer not to answer
5. Are you Hispanic / Latino?
    a. Yes
    b. No
    c. Prefer not to answer
6. How would you describe your sexual orientation?
    a. Heterosexual
    b. Bisexual
    c. Gay / Lesbian
    d. Queer
    e. Questioning
    f. Other (Please specify):
    g. Prefer not to answer
7. Do you live with any type of disability?
    a. Yes
        i. What disability do you live with?
    b. No
    c. Prefer not to answer
8. Have you received a diagnosis of a mental illness?
    a. Yes (Please specify your diagnosis):
    b. No
    c. Prefer not to answer
9. If you have not been diagnosed with a mental illness, do you believe that you have a mental illness?

    a. Yes (Please specify what mental illness you believe you are experiencing):
    b. No
    c. Not applicable (I have been diagnosed with a mental illness).
    d. Prefer not to answer
10. Have you ever seen a mental health professional for any sort of difficulties (e.g. emotional, behavioral, cognitive)?
    a. Yes (Please explain what type of professional you have seen):
    b. No
    c. Prefer not to answer
11. If you have seen a mental health professional, was this helpful in addressing your difficulties?
    a. Yes
    b. No
    c. Prefer not to answer
12. Have you ever taken a medication for your mental health?
    a. Yes (Please specify the medication):
    b. No
    c. Prefer not to answer
13. Have you ever been hospitalized for your mental health?
    a. Yes
    b. No
    c. Prefer not to answer
14. Are you currently subscribed to Cope Notes?
    a. Yes
    b. No
15. If you are still using Cope Notes, how long have you been using Cope Notes? Please specify number of days:
16. If you are no longer using Cope Notes, how long did you use Cope Notes before stopping? Please specify number of days:
17. If you are no longer using Cope Notes, why did you stop using Cope Notes?
18. Are you a current college student?
    a. Yes
        i. Are you an undergraduate student or graduate student?
            a) Undergraduate Student
            b) Graduate Student
            c) USF Morsani College of Medicine Student
    b. No
19. Are you a SELECT student?
    a. Yes
        i. What is your major?
        ii. Are you the first in your family to attend college?
            a) Yes
            b) No
            c) Prefer not to answer
    b. No

20. Would you consider the area in which you reside to be rural or urban?
    a. Rural
    b. Urban
21. Are you subscribed to Cope Notes via one of the following selections?
    a. Free trial
    b. Paid subscription
    c. Gift subscription
    d. Prefer not to answer
