# Supplementary material for: Characterizing User Experiences With an SMS Text Messaging–Based mHealth Intervention: Mixed Methods Study
Source: JMIR Form Res. 2022 May 3;6(5):e35699. doi: 10.2196/35699 (PMC9115655; doi:10.2196/35699)
Supplement: Multimedia Appendix 2 [file formative_v6i5e35699_app2.pdf]

This is a Multimedia Appendix to a full manuscript published in the JMIR Form Res. For full copyright and citation information see https:// doi.org/10.2196/35699.

Cope Notes User Experiences

Please provide the email address used to sign up for the Cope Notes study group.

Rate your level of agreement with the following statements regarding the content of Cope Notes messages received over the past 30 days.

	Not applicable	Strongly Disagree	Disagree	Somewhat Disagree	Neither agree nor disagree	Somewhat Agree	Agree	Strongly Agree
	0	1	2	3	4	5	6	7
Cope Notes messages were relevant to my life as a whole, regardless of when they were received. ()								
Cope Notes messages came at a relevant time in my life. ()								
Cope Notes messages were not relevant to my life at all. ()								
I think of Cope Notes messages often, and remembering them helps me face new situations. ()								
Cope Notes messages provoked a positive feeling. ()								
Cope Notes messages provoked a negative feeling. ()								
I fully understood the sentiment behind Cope Notes messages. ()								
Cope Note messages helped me view myself or my situation differently. ()								
Cope Notes messages helped me deal with or relieve pressure or stress. ()								
I have shared Cope Notes messages with others or posted them on a social networking site. ()								

How well do the following statements describe your personality?

I see myself as someone who...

	Not applicable	Strongly disagree	Somewhat disagree	Neither agree nor disagree	Somewhat agree	Strongly agree
... is reserved	○	○	○	○	○	○
... is generally trusting	○	○	○	○	○	○
... tends to be lazy	○	○	○	○	○	○
... is relaxed, handles stress well	○	○	○	○	○	○
... has few artistic interests	○	○	○	○	○	○
... is outgoing, sociable	○	○	○	○	○	○
... tends to find fault with others	○	○	○	○	○	○
... does a thorough job	○	○	○	○	○	○
... gets nervous easily	○	○	○	○	○	○
... has an active imagination	○	○	○	○	○	○

Read each statement carefully and indicate your degree of agreement using the scale below.

	Not applicable	Disagree	Partly disagree	Partly agree	Agree
If I believed I was having a mental breakdown, my first inclination would be to get professional attention.	○	○	○	○	○
Talking about my problems with a psychologist strikes me as a poor way to handle emotional conflicts.	○	○	○	○	○
If I were experiencing a serious emotional crisis at this point in my life, I would be confident that I could find relief in psychotherapy.	○	○	○	○	○
There is something admirable in the attitude of a person who is willing to cope with his or her conflicts and fears without resorting to professional help.	○	○	○	○	○
I would want to psychological help if I were worried or upset for a long period of time.	○	○	○	○	○
I might want to have psychological counseling in the future.	○	○	○	○	○
A person with an emotional problem is better off working through it with a professional than trying to handle it alone.	○	○	○	○	○
Considering the time and expense involved with psychotherapy, I doubt that it would be worth it for me.	○	○	○	○	○
A person should work out their own problems. Psychological counseling should be treated as a last resort.	○	○	○	○	○
Personal and emotional troubles, like many things, tend to work out by themselves.	○	○	○	○	○

Rate your level of agreement with the following statements.

	Not applicable	Strongly disagree	Disagree	Slightly disagree	Slightly agree	Agree	Strongly agree
Most people would willingly accept a former mental patient as a close friend.	○	○	○	○	○	○	○
Most people believe that a person who has been in a mental hospital is just as intelligent as the average person.	○	○	○	○	○	○	○
Most people believe that a former mental patient is just as trustworthy as the average citizen.	○	○	○	○	○	○	○
Most people would accept a fully recovered former mental patient as a teacher of young children in a public school.	○	○	○	○	○	○	○
Most people feel that entering a mental hospital is a sign of personal failure.	○	○	○	○	○	○	○
Most people would not hire a former mental patient to take care of their children, even if he or she had been well for some time.	○	○	○	○	○	○	○
Most people think less of a person who has been in a mental hospital.	○	○	○	○	○	○	○
Most employers will hire a former mental patient if he or she is qualified for the job.	○	○	○	○	○	○	○
Most employers would decline the application of a former mental patient in favor of another applicant.	○	○	○	○	○	○	○
Most people in my community would treat a former mental patient just as they would treat anyone.	○	○	○	○	○	○	○
Most young women would be reluctant to date a man who has been hospitalized for a serious mental disorder.	○	○	○	○	○	○	○
Upon finding out that someone is a former mental patient, most people will take that person’s opinions less seriously.	○	○	○	○	○	○	○

Rate each coping strategy below according to how well you feel it works for dealing with various problems. You can replace the word “it” with a relevant problem that you have or may be facing. Consider each strategy independently of the others.

	I haven't been doing this at all.	I've been doing this a little bit.	I've been doing this a medium amount.	I've been doing this a lot.
I've been turning to work or other activities to take my mind off things.	○	○	○	○
I've been concentrating my efforts on doing something about the situation I'm in.	○	○	○	○
I've been saying to myself "this isn't real."	○	○	○	○
I've been using alcohol or other drugs to make myself feel better.	○	○	○	○
I've been getting emotional support from others.	○	○	○	○
I've been giving up trying to deal with it.	○	○	○	○
I've been taking action to try to make the situation better.	○	○	○	○
I've been refusing to believe that it has happened.	○	○	○	○
I've been saying things to let my unpleasant feelings escape.	○	○	○	○
I’ve been getting help and advice from other people.	○	○	○	○
I've been using alcohol or other drugs to help me get through it.	○	○	○	○
I've been trying to see it in a different light, to make it seem more positive.	○	○	○	○
I’ve been criticizing myself.	○	○	○	○
I've been trying to come up with a strategy about what to do.	○	○	○	○
I've been getting comfort and understanding from someone.	○	○	○	○
I've been giving up the attempt to cope.	○	○	○	○
I've been looking for something good in what is happening.	○	○	○	○
I've been making jokes about it.	○	○	○	○

I've been doing something to think about it less, such as going to movies, watching TV, reading, daydreaming, sleeping, or shopping.	○	○	○	○
I've been accepting the reality of the fact that it has happened.	○	○	○	○
I've been expressing my negative feelings.	○	○	○	○
I've been trying to find comfort in my religion or spiritual beliefs.	○	○	○	○
I’ve been trying to get advice or help from other people about what to do.	○	○	○	○
I've been learning to live with it.	○	○	○	○
I've been thinking hard about what steps to take.	○	○	○	○
I’ve been blaming myself for things that happened.	○	○	○	○
I've been praying or meditating.	○	○	○	○
I've been making fun of the situation.	○	○	○	○
