# Supplementary material for: Characterizing User Experiences With an SMS Text Messaging–Based mHealth Intervention: Mixed Methods Study
Source: JMIR Form Res. 2022 May 3;6(5):e35699. doi: 10.2196/35699 (PMC9115655; doi:10.2196/35699)
Supplement: Multimedia Appendix 3 [file formative_v6i5e35699_app3.pdf]

This is a Multimedia Appendix to a full manuscript published in the JMIR Form Res. For full copyright and citation information see https://doi.org/10.2196/35699.

Minor Code Definitions

Theme	ID	Code	Definitions
Likes/Perceived Benefits	1	positive impact	positive reaction to Cope Notes
	2	reframing	thinking about cope notes
	3	convenient	passive, simple
	4	SMS engagement	willingness to respond
	5	positive delivery strategy	timing in general
	6	message content	reaction to the cope note
	7	lifestyle	Cope Notes fits into lifestyle
	8	priming	Cope Notes influences mindset on day-to-day basis
	9	breaks routine	break from lifestyle
	10	website interface	opinion on website
	11	comparison	comparison between other services, what the user preferred
Dislikes/Limitations	12	SMS engagement	lack of satisfaction with interactivity ability
	13	lack of response	one way messages
	14	lack of impact	limited outreach or stigma reduction
	15	message content	lack of satisfaction with message content
	16	negative delivery strategy	timing too late/early
	17	cost	price comparison
	18	technology glitch	service glitch
Suggested Changes	19	customized delivery strategy	variability in delivery timing
	20	SMS engagement	request for more interactivity
	21	customized message content	tailored content
	22	two way interaction	need for two way interaction
	23	clarification	lack of clarity/purpose
	24	cost	price comparison
	25	content glitch	service glitch

Stigma/Help Seeking	26	background	family/culture influence towards mental health
	27	student	experience as student influence towards mental health
	28	lived experience	lived experience with mental health
	29	stigma reduction	destigmatization of mental health
	30	help seeking	encouragement of help seeking
	31	personal stigma	personal views on stigma/mental health
	32	limited impact	limited outreach or stigma reduction
Alternative mHealth	33	app comparison	comparing Cope Notes to another app
	34	ASMR	Autonomous sensory meridian response videos
	35	other mHealth/therapy	other coping mechanisms accessible via mobile phone
	36	alternate SMS intervention	other Text message interventions
	37	journaling	coping mechanism
Perceptions of Ubiquitous Sensing	38	positive reaction	user reaction to Cope Notes extended idea
	39	negative reaction	user reaction to Cope Notes extended idea
	40	neutral reaction	lack of pos/neg reaction to Cope Notes extended idea
	41	positive sensors	specific sensors noted by participants with positive sentiments
	42	neutral sensors	specific sensors noted by participants with neutral sentiments
	43	negative sensors	specific sensors noted by participants with negative sentiments
	44	groupthink privacy concern	offering assumed views of people as a whole
	45	personal privacy concern	personal views on tech privacy
	46	unsure of tech abilities	user not confident in the ability of ML and other technologies to infer moods, user not confident in the usefulness information gathered to infer moods

	47	transparency to user	communicate clearly what aspects of which sensors is being collected, and the purpose of why these sensors are being used
	48	purpose of collection	purpose of data being collected
	49	user access to data	user ability to access data collected
	50	positive cultural impact	viewing Cope Notes as culturally accepting
	51	neutral cultural impact	felt that Cope Notes does not address cultural differences
Cultural Sensitivity	52	negative cultural impact	Cope Notes lacks in cultural sensitivity
